# Craniofacial Features in Children with Sleep-Disordered Breathing: A Comparative Study 

**DOI:** 10.30476/dentjods.2025.106103.2632

**Published:** 2026-09-01

**Authors:** Maryam Karandish, Sara Nemati, Alireza Sharifinejad, Mohammad Ashkan Moslehi

**Affiliations:** 1 Orthodontic Research Center, School of Dentistry, Shiraz University of Medical Sciences, Shiraz, Iran.; 2 Student Research committee, School of Dentistry, Shiraz University of Medical Sciences, Shiraz, Iran.; 3 Dept. of Pediatric Dentistry, School of Dentistry, Shiraz University of Medical Sciences, Shiraz, Iran.; 4 Division of Pediatric Interventional Pulmonology, Dept. of Pediatrics, School of Medicine, Shiraz University of Medical Sciences, Shiraz, Iran.

**Keywords:** Cephalometry, Obstructive sleep apnea, Sleep-disordered breathing, Pediatrics

## Abstract

**Background:**

Sleep-disordered breathing (SDB) consists of a range of symptoms that can negatively affect children's quality of life. Earlier diagnosis of SDB in children can result in the prevention of severe outcomes.

**Purpose:**

This study aimed to evaluate the craniofacial features and skeletal growth patterns of children with SDB.

**Materials and Method:**

This comparative case-control study was conducted on 150 patients aged 8 to 12 years who attended the orthodontic department. Based on the Berlin questionnaire, selected patients were divided into two groups of 25, including children with a high risk of SDB (SDB group) and those without the risk (control group). Craniofacial analysis of all patients was carried out on their lateral cephalometric radiographs. Cephalometric sagittal and vertical parameters were compared between groups using the Mann-Whitney U test with effect sizes reported. Data normality was assessed to justify statistical methods.

**Results:**

Vertical parameters, including FMA (*p* Value= 0.019, Cohen’s d= 0.69) and GoGn-SN angles (*p*= 0.038, Cohen’s d= 0.46), were significantly higher, and the Jarabak index was lower (*p*= 0.022, Cohen’s d= 0.51) in the SDB group, indicating a vertical growth pattern. However, the anterior-posterior position of both jaws was not significantly different between the two groups (*p*> 0.05).

**Conclusion:**

In this study, considerable differences were observed in the craniofacial features of children with SDB, particularly in the vertical dimension.

## Introduction

Sleep-disordered breathing (SDB) in pediatric patients encompasses a range of symptoms, including snoring, gasping, and unexplained bed wetting to more severe presentations like airway obstruction and sleep apnea [ 1 - 2 ]. This disease might affect children's quality of life in many aspects, including their behavior and school performance [ 3 - 4 ].

Although nocturnal polysomnography is regarded as the best test for the diagnosis of SDB, it is still unavailable and expensive [ 5 ]. Instead, the Berlin Questionnaire is a simple and valid screening tool used for snoring and can predict high-risk patients for developing sleep apnea [ 6 - 7 ]. A series of treatments have been recommended for SDB patients, including weight control and corticosteroids to orthognathic surgery, or tracheostomy in severe cases [ 8 ]. Besides, orthodontic appliance therapy, such as rapid maxillary expansion, has shown promise in improving airway dimensions as an adjunctive method for alleviating SDB symptoms. Earlier diagnosis of SDB tendency in children can result in the prevention of severe outcomes [ 9 ]. 

In orthodontic practice, craniofacial analysis is based on the tracing of anatomical structures on lateral cephalograms [ 10 ]. Several measurements achieved from orthodontic tracing are used for the diagnosis of skeletal discrepancies and treatment planning. Recent studies have investigated some of the craniofacial characteristics that are more prevalent in SDB patients [ 5 , 11 - 14 ]. Excessive overjet, mandibular retrognathism, increased vertical growth of the mandible, smaller cranial base angle, and smaller dimensions of the pharynx are reported to be more common in these patients [ 5 , 13 - 14 ]. On the other hand, some studies have reported no relationship between SDB and mandibular retrognathism or long-face tendency [ 15 - 17 ]. Despite the conduction of the aforementioned studies, there are still contradictory results regarding the craniofacial characteristics of SDB children [ 18 ]. Several methodological errors have been raised concerning the validity of their results. Due to low certainty of the evidence, a relationship between craniofacial morphology and pediatric SDB cannot be supported at this time [ 18 ]. Regarding multiple confounding factors that affect craniofacial development, performing well-designed studies with strict inclusion criteria is necessary in this topic [ 18 - 19 ].

This study investigated the hypothesis that long face, retrognathic face morphology of orthodontic patients will have positive correlations with their tendency to develop SDB. The aim of this study was to compare the cephalometric features of children with SDB with the control group.

## Materials and Method

This study was approved by the Ethical Committee of Shiraz University of Medical Sciences (IR.SUMS.REC. 1396.S567), and informed consent was signed by all patients and their parents/official guardians. Among patients between the ages of 8 to 12 years who attended the orthodontic department of Shiraz dental school, 150 patients were selected through an accessible sampling method. The inclusion criteria of this study were: 1) children between 8 to 12 years old, 2) having between 20 to 30kg weight, 3) with an indication for orthodontic treatment, and 4) without a history of systemic diseases. Taking radiographs from the normal population is not possible due to ethical issues [ 20 ]; therefore, patients in our study were chosen from orthodontic patients with a need for lateral cephalograms. Exclusion criteria consisted of: 1) any acute ear, nose, and throat infections, 2) asthma or allergies, 3) recurrent infection of lungs or sinuses, 4) post-nasal dripping, 5) skeletal class III anomaly, and 6) history of previous orthodontic treatment. 

 The Berlin questionnaire was asked of parents of these patients under the supervision of a pediatric pulmonologist [ 7 ]. Patients were considered to be at high risk for SDB if at least two of the three categories were scored positive. On the other hand, low-risk patients were defined if fewer than two categories were positive. Based on the responses given to the questionnaire, 34 patients out of 150 children were diagnosed as being at high risk of SDB. High-risk patients were then referred to the pediatric pulmonologist for further examination through polysomnography. An apnea/hypopnea index greater than 5/hrs in polysomnography was regarded as confirmation of SDB. Finally, 25 children were diagnosed with SDB. Among the 116 participants diagnosed to be at low risk of sleep apnea, 25 patients were randomly selected for the control group. The randomization was performed through computer-generated list of numbers. Hence, this study consisted of two groups (n=25/group) including a group with high risk of SDB (SDB group) and a low-risk group (control group). 

In order to investigate other confounding factors in this study, history of parental smoking, breastfeeding, oral habits, and preterm delivery were also recorded.

To evaluate the craniofacial features, orthodontic tracing was done manually on patients’ standard lateral cephalometric radiographs by an experienced orthodontist. All lateral cephalometric radiographs were taken by the same X-ray machine (Carestream ^TM^, Georgia, USA). The orthodontist was blinded about each patient's group while carrying out the cephalometric analysis. Subsequently, sagittal and vertical dimension analysis were performed and compared between the two groups. In order to evaluate the intra-examiner variability of the examiner, 10 randomly selected radiographs were analyzed twice by the orthodontist with an interval period of 1 month. Then the intra-examiner variability was assessed through Cohen's kappa coefficient. 

The skeletal parameters measured in this study were as follows:

*Sagittal dimension:* SNA (anteroposterior position of the maxilla relative to the anterior cranial base), SNB (anteroposterior position of the mandible relative to the anterior cranial base), ANB (mandibular anteroposterior position in relation to that of maxilla) and Wits (millimeter distance between lines Perpendicular to points A and B on functional occlusal planes). 

*Vertical dimension:* Jarabak index (the ratio of posterior facial height (S-Go) to anterior facial height (N-Me)), FMA (The angle between the mandibular plane and Frankfurt Plane), GoGn-SN (The angle between the mandibular plane and anterior cranial base (SN)), and inclination angle (basal maxillary tendency of maxilla to the base of the skull). 

The description of the data was done using the mean ±SD. The comparison between the two groups was done using the Mann-Whitney nonparametric test. Normality was assessed using the Shapiro-Wilk test, justifying the use of the Mann-Whitney U test for non-normally distributed data. Effect sizes (Cohen’s d) were calculated for significant differences. Comparisons were performed using SPSS software (version 25). A *p*< 0.05 was considered significant. 

## Results

This study was conducted on 50 patients with a mean age of 9.6 (±1.5) years old (23 girls and 27 boys). Age, gender, and weight measurements were not significantly different between the two groups (Table 1). All the participants were from the same ethnicity. 

**Table 1:** Characteristics of patients participating in this study

Group	SDB	Control	*p* Value
N	25	25	
Age, Mean±SD	9.3±1.2	9.8±1.44	1.00
Weight (kg), Mean±SD	24.8±2.6	27.3±1.9	0.294
Gender, n (%)		
Male	12 (48%)	15 (60%)	0.172
Female	13 (52%)	10 (40%)	

SDB: Sleep-disordered breathing

Oral habits in the SDB group were significantly greater than the control group (p: 0.049) (Table 2). However, there was no significant relationship between parental smoking, breastfeeding, and preterm deliveries with SDB.

**Table 2:** Comparison of nonparametric variables in SDB and control groups

Group	SDB	Control	*p* Value
Parental smoking	6	3	0.54
Preterm delivery	1	1	1.00
Oral habits	7	1	0.049*
Breast feeding	17	20	0.76

SDB: Sleep-disordered breathing, * statistically significant

**Figure 1:**
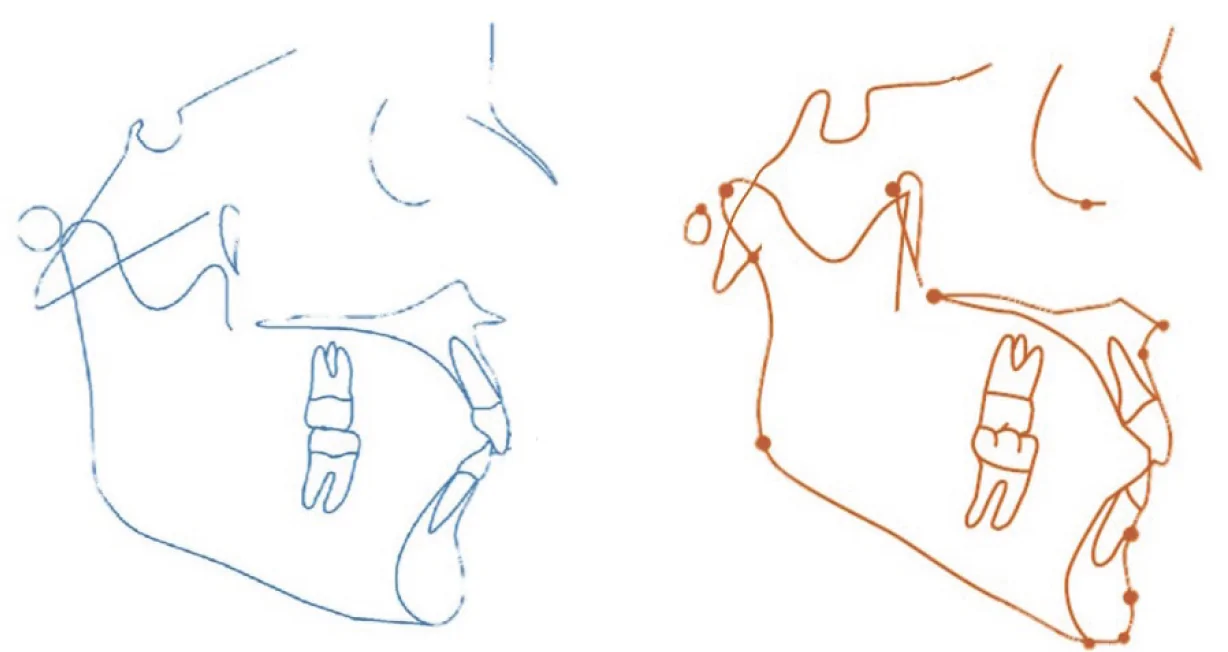
Cephalometric tracing of two patients, illustrating long face tendency in a patient with history of sleep apnea (Right-red), and short face pattern in another patient with low risk of sleep disordered breathing (Left-blue). The anterior-posterior skeletal and dental pattern of both patients shows Class I relationship

Although SDB children show a slight tendency to skeletal retrognathia, no significant differences were observed in the SNA, SNB, ANB, Wits, and inclination angle between the SDB and control groups (Table 3). The value of the Jarabak index in the SDB group was significantly lower than in the control group (*p* Value: 0.022). Both the FMA and GoGn-SN angles were significantly higher in the SDB group than the control group (*p*: 0.019 and 0.038, respectively). The aforementioned measurements illustrate that patients with SDB have a greater tendency to long-face anatomy (Figure 1). 

**Table 3:** Comparison of cephalometric measurements in SDB and control groups

Variable	SDB group (n=25) Mean±SD	Control group (n=25) Mean±SD	Mann- Whitney test *p* Value	Cohen’s d
SNA	81.8±2.79	82.04±3.75	0.792	-
SNB	77.36±1.97	78.3±4.00	0.394	-
ANB	4.44±3.04	3.74±2.06	0.352	-
Wits	3.04±1.14	2.56±1.00	0.079	-
Jarabak index	62.96±2.00	64.14±2.60	0.022*	0.51
FMA	29.68±3.72	26.88±4.23	0.019*	0.69
GoGn-SN	35.52±4.43	33.56±3.99	0.038*	0.46
Inclination angle	86.28±2.63	85.6±3.24	0.278	-

SDB: Sleep-disordered breathing, * statistically significant.

The intraclass correlation coefficient (ICC) for cephalometric measurements was 0.94, indicating high intra observer reliability.

## Discussion

Despite the available evidence investigating the craniofacial features of children with sleep-disordered breathing in different ages and communities, contradictory results can be seen due to the lack of well-designed studies [ 18 , 21 - 24 ]. The purpose of this study was to compare some of the measurable parameters in cephalometric radiographs in children with SDB with the control group.

Identifying SDB patients in the study of Katayal *et al*. [ 21 ] was solely based on parents' answers to the Berlin questionnaire. Some studies have shown that parental reports of their child's symptoms have been exaggerated [ 22 - 23 ]. Therefore, in the present study, patients who were likely to develop SDB according to the Berlin questionnaire were referred for further confirmation of diagnosis to the pediatric pulmonologist. The sample population are selected among school-aged children, due to the high prevalence of SDB in this age and feasibility of orthodontic treatments for skeletal discrepancies.

The results of this study did not show a significant difference between the SDB group and control group in the measurements of sagittal dimension (SNA, SNB, ANB, and Wit's). The absence of statistically significant sagittal skeletal differences in our study is inconsistent with studies that reported children with SDB showed an increased ANB angle caused by mandibular retrognathia by a clinically significant value of 1.6 when compared with the controls [ 5 , 16 , 19 ]. 

 According to the present study, vertical dimension parameters including the FMA and GoGn-SN angles were significantly greater in the SDB group; indicating longer lower facial height and clockwise rotation of the mandible in the SDB patients. In a systematic review and meta-analysis performed by Katayal *et al*. [ 5 ], the mandibular plane angle was greater in children with sleep-disordered breathing, which indicates longer facial height in these children than that of the control group. The findings of the meta-analysis performed by Flores Mir *et al*. [ 16 ] are also consistent with our study regarding vertical features. The study of Galeotti *et al*. [ 24 ] has concluded that vertical jaw discrepancy of SDB patients correlates with the severity of their sleep apnea. The moderate effect sizes (Cohen’s d= 0.46-0.69) suggest clinical relevance, warranting further investigation into their impact on airway obstruction. 

In the study of Parkinen *et al*. [ 15 ], like our study, the SN-Mandibular plane was significantly higher in the SDB group than in the control group. The study of Xu *et al*. [ 25 ] also showed a higher gonial angle in relation to the apnea-hypopnea index. However, the result of the study by Lee *et al*. [ 26 ] were inconsistent with these results and there was no significant difference in the mandibular plane angle between children with sleep apnea and control group. This discrepancy may stem from population-specific factors (e.g., ethnic variations in craniofacial morphology and age). While Lee *et al*. [ 26 ] has evaluated the SDB in the preschool children, the population in the study of Parkinen [ 15 ], Xu [ 25 ], and the current study are among the school aged children.

The results of this study did not show a significant difference in the inclination angle between the case and the control group. Determination of the maxillary rotation has not been considered in previous studies, instead the angle between palatal and mandibular planes has been measured in order to evaluate the skeletal facial divergence. In the study of Katayale *et al*. [ 21 ], the angle between the palatal planes and the SN line was measured to examine the maxillary rotation, and no significant difference between patients with SDB and the control group was found.

In our study, there was a significant higher prevalence of oral habits history in children and the development of SDB. Similarly, a study by Sabuncuoglu [ 27 ] has stated that the habit of thumb-sucking in children leads to malocclusion which can predispose children to SDB. It has been reported that oral functions are significantly correlated with nasal obstruction [ 28 ].

The results of current study showed no significant relationship between parental smoking and SDB which confirmed the results of Banabilh *et al*. [ 13 ]. There was no significant relationship between children birth and preterm delivery with SDB. However, the results of the study by Walfisch *et al*. [ 29 ] showed that preterm children had a higher chance of developing Sleep apnea syndrome. Also, there was no significant relationship between breastfeeding and SDB in children in the present study. In a systematic review and meta-analysis by Garcia *et al*. [ 30 ] that investigated the relationship between breastfeeding and SDB In children, most included studies have shown that breastfeeding reduces the likelihood of SDB. Confounding factors might play a role in the differences seen between studies. 

From a clinical perspective, orthodontists can play a critical role in early SDB identification by screening for vertical craniofacial patterns and oral habits. Treatments like rapid maxillary expansion may improve airway dimensions and SDB symptoms, as supported by Katyal *et al*. [ 21 ]. Long-term SDB consequences, including cognitive deficits, behavioral issues, and cardiovascular risks, underscore the need for early intervention. Interdisciplinary collaboration between orthodontists, pediatric pulmonologists and sleep specialists is essential for comprehensive management [ 31 ].

Due to the ethical limitation of not exposing healthy children to X-ray radiation, the control group of this study was selected among orthodontic patients; though our results cannot be generalized to the normal population. Further research with a larger sample size, multicenter approach, and more generalized subjects is recommended. Longitudinal studies are needed to establish causal relationships between craniofacial features and SDB, and advanced imaging techniques such as cone beam computed tomography (CBCT) could provide deeper insights into airway morphology [ 32 ].

## Conclusion

In the present study, considerable differences were observed in the craniofacial features of children with SDB, particularly in the vertical dimension. Dentists should be aware of the craniofacial features that are associated with sleep breathing disorders, to help timely diagnosis of these patients, and appropriate treatment plans to improve patient's quality of life.
